# Expression Profiling during Arabidopsis/Downy Mildew Interaction Reveals a Highly-Expressed Effector That Attenuates Responses to Salicylic Acid

**DOI:** 10.1371/journal.ppat.1004443

**Published:** 2014-10-16

**Authors:** Shuta Asai, Ghanasyam Rallapalli, Sophie J. M. Piquerez, Marie-Cécile Caillaud, Oliver J. Furzer, Naveed Ishaque, Lennart Wirthmueller, Georgina Fabro, Ken Shirasu, Jonathan D. G. Jones

**Affiliations:** 1 The Sainsbury Laboratory, Norwich Research Park, Norwich, United Kingdom; 2 Center for Sustainable Resource Science, RIKEN, Tsurumi, Yokohama, Kanagawa, Japan; 3 John Innes Centre, Norwich Research Park, Norwich, United Kingdom; University of North Carolina at Chapel Hill, United States of America

## Abstract

Plants have evolved strong innate immunity mechanisms, but successful pathogens evade or suppress plant immunity via effectors delivered into the plant cell. *Hyaloperonospora arabidopsidis* (*Hpa*) causes downy mildew on *Arabidopsis thaliana*, and a genome sequence is available for isolate Emoy2. Here, we exploit the availability of genome sequences for *Hpa* and Arabidopsis to measure gene-expression changes in both *Hpa* and Arabidopsis simultaneously during infection. Using a high-throughput cDNA tag sequencing method, we reveal expression patterns of *Hpa* predicted effectors and Arabidopsis genes in compatible and incompatible interactions, and promoter elements associated with *Hpa* genes expressed during infection. By resequencing *Hpa* isolate Waco9, we found it evades Arabidopsis resistance gene *RPP1* through deletion of the cognate recognized effector *ATR1*. Arabidopsis salicylic acid (SA)-responsive genes including *PR1* were activated not only at early time points in the incompatible interaction but also at late time points in the compatible interaction. By histochemical analysis, we found that *Hpa* suppresses SA-inducible *PR1* expression, specifically in the haustoriated cells into which host-translocated effectors are delivered, but not in non-haustoriated adjacent cells. Finally, we found a highly-expressed *Hpa* effector candidate that suppresses responsiveness to SA. As this approach can be easily applied to host-pathogen interactions for which both host and pathogen genome sequences are available, this work opens the door towards transcriptome studies in infection biology that should help unravel pathogen infection strategies and the mechanisms by which host defense responses are overcome.

## Author Summary

A comprehensive understanding of host-pathogen interactions requires knowledge of the dynamics of gene expression changes in both the host and the pathogen during a time course of infection. However, expression profiling has often focused on either the host or the pathogen due to limitations of methods that involve microarrays. We report here gene expression changes in both Arabidopsis and its parasite *Hyaloperonospora arabidopsidis* (*Hpa*) simultaneously during infection using a high-throughput RNA sequencing method. By resequencing *Hpa* isolate Waco9, we found it evades Arabidopsis resistance gene *RPP1* through deletion of cognate recognized effector *ATR1*. We also found that *Hpa* suppresses responsiveness to salicylic acid (SA) in haustoriated cells into which host-translocated effectors are delivered. An *Hpa* effector *HaRxL62*, previously shown to enhance host susceptibility, was highly expressed in this assay, and we found it suppresses responsiveness to SA. Expression profiling of both pathogen effector genes and host genes involved in immunity allows us to suggest distinct mechanisms of effector-mediated susceptibility and reveals interesting *Hpa* effectors for detailed mechanistic investigation in future experiments.

## Introduction

During co-evolution with pathogens, plants have evolved multiple immune signaling mechanisms that successful pathogens have evolved to evade or suppress. The first layer is based on recognition of broadly conserved pathogen molecules (pathogen/microbe-associated molecular patterns, PAMP/MAMPs) by plant cell surface pattern-recognition receptors (PRRs), resulting in PAMP- (or pattern)-triggered immunity (PTI) [1]. However, PTI can be suppressed by pathogen proteins, termed effectors, that are delivered into the apoplast or plant cell cytoplasm, resulting in effector-triggered susceptibility. Plants also carry a second layer of defense, so-called effector triggered immunity (ETI), in which cytoplasmic disease resistance (R) proteins recognize directly or indirectly the presence of pathogen effectors. Recognized effectors are often known as avirulence (AVR) proteins [2], [3]. A hallmark of ETI is the hypersensitive response (HR), which involves programmed cell death at pathogen infection sites and helps resist biotrophic pathogens.

In many oomycetes, such as *Phytophthora* spp. and downy mildews, the most common host-translocated effectors are the RxLR-type proteins that contain an N-terminal signal peptide and a RxLR (or RxLR-EER) motif involved in secretion and host uptake, and a C-terminal domain carrying the effector activity [3]–[5]. *Hyaloperonospora arabidopsidis* (*Hpa*; formerly *Peronospora parasitica* or *Hyaloperonospora parasitica*) is an obligate biotrophic oomycete that causes downy mildew in *Arabidopsis thaliana*. The Arabidopsis-*Hpa* pathosystem has been extensively used to study host/pathogen co-evolution, and has enabled identification of cognate host *R* and pathogen *AVR* genes, termed *RPP* (recognition of *Peronospora parasitica*) and *ATR* (*Arabidopsis thaliana* recognized), respectively [6]. Genome analysis of *Hpa* isolate Emoy2 identified 134 high-confidence effector candidates (HaRxL genes) [7]. Comprehensive screening of HaRxL effectors revealed that the majority of HaRxLs contribute positively to pathogen fitness [8], [9]. In addition, HaRxLs can be located in different subcellular compartments *in planta* [10]. Some have been shown by yeast two hybrid screens to interact with various plant proteins [11]. However, the mechanisms by which most *Hpa* effectors promote virulence remain to be elucidated.

Salicylic acid (SA) is a phytohormone essential for the immune response against biotrophic pathogens [12]. SA biosynthesis is triggered during both PTI and ETI [13]. Signaling downstream of SA is largely controlled by the regulatory protein NON-EXPRESSOR OF PR GENES1 (NPR1), which upon activation by SA acts as a transcriptional coactivator of a large set of defense-related genes, such as *PATHOGENESIS-RELATED GENE 1* (*PR1*) [14]. Another phytohormone, jasmonic acid (JA), is synthesized upon pathogen and herbivore attack, and is essential for the immune response against necrotrophic pathogens and herbivores [15]. Multiple studies revealed a mutually antagonistic interaction between SA- and JA-dependent signaling [16], [17]. Some pathogens and herbivores appear to induce SA-JA crosstalk [18]–[23]. For example, *Pseudomonas syringae* produces coronatine, a toxin that mimics the bioactive jasmonate JA-isoleucine [24] and promotes stomatal reopening and bacterial propagation in both local and systemic tissues by inhibiting SA signaling and accumulation [20], [23]. In addition to SA and JA, recent studies have revealed involvement of other phytohormones, such as ethylene (ET), abscisic acid (ABA), gibberellin and auxin, in biotic interactions [25]. Remarkably, several pathogens produce phytohormones and phytohormone mimics like coronatine in *P. syringae*.

To dissect the Arabidopsis-*Hpa* interaction, changes in expression of Arabidopsis or *Hpa* genes during infection were previously investigated by microarray analysis for Arabidopsis genes [26]–[29] and by cDNA-amplified fragment length polymorphism and expressed sequence tag analysis for *Hpa* genes [30]–[32]. In *Hpa*, however, these approaches were not sensitive enough to enable genome-wide quantification of changes in gene expression during infection. Expression profiling in Arabidopsis or *Hpa* was carried out with different Arabidopsis accessions, *Hpa* isolates, plant ages and infection time courses, hindering comparison of these data. Recently, we established a high-throughput mRNA expression-profiling method (Expression Profiling through Random Sheared cDNA tag Sequencing [EXPRSS]) enabling the detection of differential expression of more genes, with higher sensitivity, than microarray and traditional RNA sequencing methods [33]. Briefly, EXPRSS is a restriction enzyme-independent tag-sequencing method and generates one tag per transcript at a relatively defined position from the 3′ end of a gene, ensuring no length-based data transformation and enabling expression data to be obtained at a ∼10× greater read depth than standard Illumina RNA sequencing. This is helpful when we investigate low-level transcripts, such as pathogen transcripts in host-pathogen interactions. Using EXPRSS, we monitored mRNA levels for both Arabidopsis and *Hpa* genes during infection. Here, we report the expression patterns of *Hpa* predicted effectors and Arabidopsis genes on the basis of transcriptome data in Arabidopsis Col-0 inoculated with the avirulent *Hpa* isolate Emoy2 (recognized by *RPP4* [34]) or the virulent isolate Waco9. From this analysis, we found that *ATR1* (recognized by *RPP1* [35]) is not expressed in *Hpa* Waco9, and after resequencing the Waco9 genome, we found the *ATR1* region is deleted. An *Hpa* effector *HaRxL62*, previously shown to enhance host susceptibility [8], [9], was highly expressed in this assay, and was shown here to suppress responsiveness to SA.

## Results

### Expression profiling of host and pathogen during Arabidopsis-*Hpa* interaction

**Figure 1:**
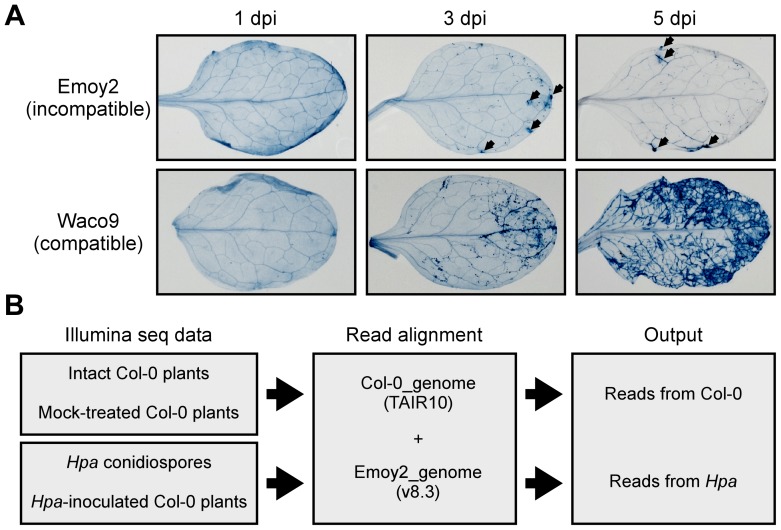
*Hpa* development and scheme for aligning Illumina sequence reads. (A) Trypan blue staining in three-week-old Arabidopsis Col-0 plants at 1, 3 and 5 dpi with *Hpa* Emoy2 and Waco9. Black arrows indicate the parts in which HR cell death was observed. (B) Work-flow scheme to separate Illumina sequencing reads from Arabidopsis and *Hpa*.

Arabidopsis Col-0 was inoculated with either the avirulent isolate Emoy2 (incompatible interaction) or the virulent isolate Waco9 (compatible interaction) of *Hpa*, and infected plants were harvested at 1, 3 and 5 days post-inoculation (dpi) prior to Illumina sequencing using EXPRSS [33]. *Hpa* haustoria are formed in both compatible and incompatible interactions till 1 dpi, and HR cell death is observed only in incompatible interactions [36]. HR was observed in *Hpa* Emoy2-inoculated leaves of Col-0 from 3 dpi, whereas no visible HR was observed at 1 dpi (Figure 1A). After *Hpa* Waco9 inoculation, extensive growth of intercellular mycelium was evident on leaves from 3 dpi, and then sporulation (conidiophores bearing conidiospores) was observed at 5 dpi (Figure 1A). In addition to the infectious stages, samples were taken from intact plants (0 dpi) and water-sprayed (mock-treated) plants as control samples for transcriptome analysis in Arabidopsis. Further, to evaluate the expression pattern of *Hpa* genes, samples were taken from conidiospores before inoculation. The experiment was carried out with three independent biological replicates.

Total RNA was prepared from infected plants, and libraries for EXPRSS were prepared. Although 36 bp sequencing reads are sufficient to identify Arabidopsis genes distinctly using EXPRSS [33], longer sequencing reads (80 bp) were used in this study to avoid cross-mapping to the Arabidopsis and *Hpa* genomes. The Illumina sequencing reads were mapped to the combined genome of Arabidopsis TAIR10 and *Hpa* Emoy2 v8.3 [7] (Figure 1B). Mapped-reads to Arabidopsis and *Hpa* genomes were counted separately and the distribution of mean expression of each gene was represented as TPM (tags per million) of total reads mapped to Arabidopsis or *Hpa* genomes. To provide sufficient depth for expression analysis of *Hpa* genes in infected plants, Illumina sequencing was carried out twice for the incompatible interaction (*Hpa* Emoy2-inoculated plants) and for the early time point (at 1 dpi) of the compatible interaction (*Hpa* Waco9-inoculated plants). In this study, we did the analyses using uniquely mapped or up to 10 matching reads (Table S1 and Datasets S1, S2 and S3; see Materials and Methods). Using only uniquely mapped reads would give a minimum estimate of high confidence in gene expression, but we might even discard the information for homologous genes. Although we cannot rule out the presence of some false positives and false negatives in the data using up to 10 matching reads, the data would contain more information including homologous genes. For these reasons, the data with up to 10 matching reads were used in the following analyses. Most reads in intact and mock-treated plants were mapped to the Arabidopsis genome (i.e. % *Hpa* reads <0.005), whereas most reads from *Hpa* conidiospores were mapped to the *Hpa* genome (i.e. % *Hpa* reads >91.7) (Table 1 and Figure S1). The reads mapped to the Arabidopsis genome in samples from *Hpa* conidiospores are likely to be due to Arabidopsis contamination in the spore inoculum, as *Hpa* was propagated on susceptible Arabidopsis accessions and its conidiospores were collected from infected Arabidopsis leaf tissues. The results suggest high gene-identification accuracy between Arabidopsis and *Hpa* in this study.

**Table 1:** Summary of transcriptome data in Arabidopsis inoculated with *Hpa*.

	Mock				Emoy2 (incompatible)				Waco9 (compatible)			
	0 dpi	1 dpi	3 dpi	5 dpi	cs	1 dpi	3 dpi	5 dpi	cs	1 dpi	3 dpi	5 dpi
Total reads assigned to Arabidopsis/*Hpa* genes	21,407,400	12,205,405	18,290,751	19,758,870	6,659,454	38,982,617	31,283,609	34,297,772	9,490,478	37,603,037	17,802,259	18,734,995
Arabidopsis reads	21,406,773	12,204,838	18,290,307	19,758,300	549,559	38,949,270	31,275,305	34,290,254	449,313	37,592,927	17,682,677	18,462,979
Expressed Arabidopsis transcripts	23,371	23,072	23,363	23,630	16,609	24,802	24,170	24,497	20,898	24,477	23,516	23,917
*Hpa* reads	626	566	444	570	6,109,895	33,347	8,304	7,518	9,041,165	10,110	119,581	272,016
Expressed *Hpa* transcripts	108	117	79	139	11,369	5,415	2,101	2,052	11,626	2,293	6,858	8,794
Expressed transcripts of predicted effectors	2	2	3	3	355	130	51	46	359	70	202	252
*Hpa* reads (%)	<0.005	<0.005	<0.005	<0.005	91.748	0.086	0.027	0.022	95.266	0.027	0.672	1.452

cs, conidiospores.

In the incompatible interaction, the number of *Hpa* reads clearly decreased from 1 dpi, whereas the population of *Hpa* reads increased in the compatible interaction (Figure S1 and Table 1). This indicates that *Hpa* Emoy2 dies upon recognition after 1 dpi, corresponding to visible HR from 3 dpi with Emoy2 (Figure 1A). Hence, the data at 3 and 5 dpi with Emoy2 were omitted from the *Hpa* transcriptome data. The analysis of the overall transcriptome data revealed that out of 27,416 protein coding genes in Arabidopsis TAIR10 and 14,489 genes in *Hpa* v8.3, 24,559 (89.6%) for Arabidopsis and 11,394 (78.6%) and 11,690 (80.7%) for *Hpa* Emoy2 and Waco9, respectively, were expressed in at least one of the samples (Table 2 and Datasets S1, S2 and S3).

**Table 2:** The number of genes detected in this study.

Arabidopsis	This study	TAIR10
Total	27,777	33,602
protein coding	24,559	27,416
pseudogenes/TE	2,203	4,827
ncRNAs	1,015	1,359
Emoy2	This study	v8.3^b^
Total (protein coding)	11,394	14,489
predicted effectors	355 (130^a^)	475
Waco9	This study	v8.3^b^
Total (protein coding)	11,690	14,489
predicted effectors	366 (277^a^)	475

a Number of genes detected during infection.

b The latest version of *Hpa* genome v8.3 (v8.3 v3).

TE, transposable element.

### Expression pattern of *Hpa* predicted effectors during infection

The *Hpa* Emoy2 genome analysis revealed 134 high-confidence effector candidates (HaRxLs) with a signal peptide and canonical RxLR (or RxLR-EER) motif [7]. These include effector candidates HaRxL17, HaRxL44 and HaRxL96 [10], [18], [37] and avirulent effectors ATR1, ATR13 and ATR39 [35], [38], [39]. ATR5 containing a signal peptide and canonical EER motif, but not a canonical RxLR motif, was identified as an avirulence gene recognized by RPP5 [40]. This report suggests the existence of effector candidates without canonical RxLR motif. In our study, we defined a total of 475 genes as predicted effectors (Table S2). The selection criteria for predicted effectors were the following: (1) high-confidence effector candidates (HaRxLs), (2) RxLR-like genes with at least one non-canonical feature, as for ATR5 (HaRxLLs), (3) putative Crinkler-homologous genes with RxLR motif (HaRxLCRNs) [4], (4) homologous genes based on amino acid sequence similarity over the 5′ region including a signal peptide and RxLR motif (e.g. HaRxL1b).

**Figure 2:**
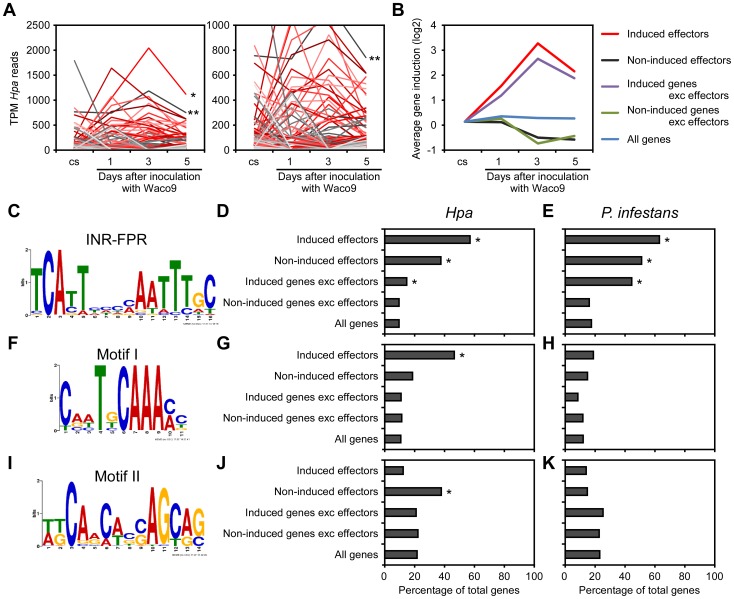
Expression pattern of *Hpa* predicted effectors and potential *cis*-regulatory elements in *Hpa*. (A) Expression pattern of predicted effectors expressed in at least one of three infections (1, 3, and 5 dpi) with *Hpa* Waco9. Expression levels were represented as TPM (tags per million) of total reads mapped to *Hpa* genome. Red lines indicate predicted effectors induced more than two fold at 3 dpi compared to the expression level in conidiospores (cs). Single and double asterisks indicate expression pattern of *HaRxL76* and *HaRxL62*, respectively. A right line chart is magnification of left one. (B) Average expression pattern of genes in the indicated groups during the infection with *Hpa* Waco9. The induction levels compared to the level in cs were indicated by value of log_2_. (C to K) Distribution of motifs in coexpressed genes of *Hpa* and *P. infestans*. Nucleotide conservation of (C) the INR-FPR motif in “induced effectors”, (F) Motif I in “induced effectors” and (I) Motif II in “non-induced effectors” is displayed as sequence logos, based on hits within 200 nt (INR-FPR) and 500 nt (Motif I and II) upstream of the start codon. Bar charts indicate the percent of promoters within each group that contain (D, E) the INR-FPR motif within 200 nt and (G, H) Motif I and (J, K) Motif II within 500 nt upstream of the start codon. The analysis was done in promoters from (D, G, J) *Hpa* and (E, H, K) *P. infestans*. Asterisks indicate statistically significant over-representation of the motifs compared to population in “all genes” (*p*<1e-4), which is shown in Table S4.

Transcriptome analysis of the compatible interaction revealed that 277 predicted effectors were expressed in at least one infection time point (Table 2). By quantifying the expression level, we found predicted effectors expressed highly during infection, e.g. HaRxL76 and HaRxL62 (about 0.2% and 0.1% of total *Hpa* mRNA at 3 dpi, respectively). In addition, most of the highly-expressed predicted effectors were upregulated more than two fold at 3 dpi compared to the expression level in conidiospores (Figure 2A). These findings suggested specific regulation of expression of some predicted effector genes upregulated at 3 dpi. To predict potential *cis*-regulatory elements in the upstream regions of *Hpa* genes, we categorized genes into five groups as follows; 87 predicted effectors which were induced more than two fold at 3 dpi (induced effectors), 115 predicted effectors which were detected at 3 dpi but were not induced more than two fold at 3 dpi (non-induced effectors), 1,880 genes excluding predicted effectors which were induced more than two fold at 3 dpi (induced genes exc effectors), 4,776 genes excluding predicted effectors which were detected at 3 dpi but were not induced more than two fold (non-induced genes exc effectors), and 14,489 genes predicted in *Hpa* v8.3 (all genes) (Table S3). The expression pattern of “induced effectors” and “non-induced effectors” was similar to “induced genes exc effectors” and “non-induced genes exc effectors”, respectively (Figure 2B). The sets of promoters of “induced effectors” and “non-induced effectors” were searched separately for conserved motifs using MEME [41], and then the motifs found were evaluated for over-representation in other groups using FIMO [42]. The INR-FPR motif, known as a core promoter element in oomycete genes [43], [44], was over-represented within 200 nt upstream of the start codon of “induced effectors” (E-value = 9.3e-068) (Figure 2C and D). The motif was also significantly over-represented in “non-induced effectors” and “induced genes exc effectors” (Figure 2D and Table S4), suggesting that INR-FPR motif is enriched in promoters of predicted effectors and genes induced during infection in *Hpa*. We also found two novel motifs (Motif I and II) within 500 nt upstream of the start codon that do not show any significant similarity to known motifs as determined by a TOMTOM search against the JASPAR database [45]. Interestingly, Motif I was overrepresented in only “induced effectors” (E-value = 8.0e-003), whereas Motif II was overrepresented in only “non-induced effectors” (E-value = 1.1e-003) (Figure 2F, G, I and J). The results suggest that Motif I and II might play a role in the regulation of the expression of predicted effector genes in *Hpa*.

To evaluate whether these motifs are conserved in other oomycetes, we checked the presence of these motifs in promoters of *Phytophthora infestans* genes co-expressed during infection according to microarray data [46]. As reported previously [43], [44], INR-FPR was over-represented in *P. infestans* RxLR effectors and genes induced during infection as observed for *Hpa* (Figure 2E and Table S4). Motif I and Motif II were not significantly over-represented in promoters of *P. infestans* genes (Figure 2H and K), suggesting that these novel motifs might be *Hpa*-specific *cis*-regulatory elements.

### *Hpa* Waco9 overcomes RPP1-mediated resistance through deletion of *ATR1*

**Figure 3:**
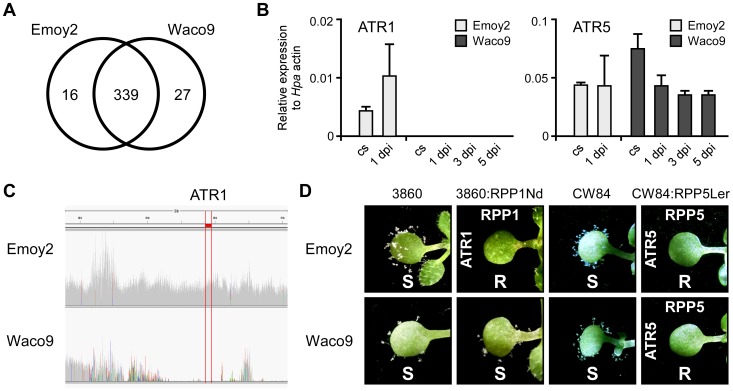
*Hpa* Waco9 overcomes recognition by RPP4, but not RPP5. (A) The number of predicted effectors expressed in *Hpa* Emoy2 and/or Waco9. (B) Expression of *ATR1* and *ATR5* in *Hpa* Emoy2 and Waco9 conidiospores (cs) and the infections in Arabidopsis Col-0. The expression level was determined by qRT-PCR using specific primers for *ATR1* and *ATR5*. Expression of *Hpa* actin was used to normalize the expression value in each sample. Data are means ± SDs from three biological replicates. (C) Illumina sequencing reads coverage in genomic region including *ATR1*. Region indicated in red is of *ATR1*. (D) Resistance (R) and susceptibility (S) to *Hpa* Emoy2 and Waco9 in seven-day-old 3860, *RPP1-Nd*-transformed 3860 (3860:RPP1Nd), CW84 and *RPP5-Ler*_transformed CW84 (CW84:RPP5Ler) plants. The plants inoculated with *Hpa* Emoy2 and Waco9 were photographed at 6 dpi.

Transcriptome analysis revealed that 355 and 366 predicted effectors were expressed in conidiospores and/or infections with *Hpa* Emoy2 and Waco9, respectively (Table 2). Of these, 339 predicted effectors were expressed in both *Hpa* Emoy2 and Waco9, whereas 16 and 27 predicted effectors were expressed in only *Hpa* Emoy2 and Waco9, respectively (Figure 3A and Table S5). *ATR5*, an effector recognized by RPP5 [40], was found among the 339 predicted effectors expressed in both *Hpa* Emoy2 and Waco9 (Figure 3B and Table S5). The Waco9 allele of *ATR5* is identical to the Emoy2 allele. Surprisingly, while *ATR1* was expressed in *Hpa* Emoy2, no tag corresponding to *ATR1* in *Hpa* Waco9 was detected (Figure 3B and Table S5). We resequenced *Hpa* Waco9 genome using an Illumina Genome Analyzer II, and found that the genomic region that includes *ATR1* is deleted in Waco9 (Figure 3C). These results suggest that *Hpa* Waco9 can infect plants containing functional *RPP1*, but not plants containing functional *RPP5*. To evaluate this possibility, several Arabidopsis accessions were inoculated with *Hpa* Emoy2 and Waco9. ATR1 from *Hpa* Emoy2 is recognized by RPP1-Nd from Arabidopsis Nd-1 accession and RPP1-WsA and RPP1-WsB from Arabidopsis Ws-2 accession (the accession previously reported as Ws-0 in our laboratory is in fact Ws-2) [35]. As expected, Arabidopsis Nd-1 and Ws-2 are resistant to *Hpa* Emoy2, but susceptible to *Hpa* Waco9 (Figure S2). We also checked the phenotype on an Arabidopsis RIL 3860 (3860), a recombinant inbred line from a cross between Col-5 and Nd-1 that lacks *RPP1-Nd*, and a transgenic 3860 line containing the functional *RPP1-Nd* gene (3860:RPP1Nd) [35]. Like Arabidopsis Nd-1 and Ws-2, 3860:RPP1Nd is resistant to *Hpa* Emoy2, but susceptible to *Hpa* Waco9, whereas Arabidopsis 3860 is susceptible to both *Hpa* Emoy2 and Waco9 (Figure 3D). On the other hand, no *Hpa* sporulation was observed on Arabidopsis Ler-0 accession containing functional *RPP5*, *RPP5-Ler*, inoculated with *Hpa* Emoy2 and Waco9 (Figure S2). To confirm if *Hpa* Emoy2 and Waco9 are recognized by RPP5-Ler, Arabidopsis CW84, a broadly *Hpa*-susceptible recombinant inbred line generated from a cross between Col-0 and Ws-2 [47], and CW84 transformants containing *RPP5-Ler* (CW84:RPP5Ler) [40] were inoculated with *Hpa* Emoy2 and Waco9. Like Arabidopsis Ler-0, CW84:RPP5Ler is resistant to both *Hpa* Emoy2 and Waco9, whereas Arabidopsis CW84 is susceptible to both *Hpa* isolates (Figure 3D). These results indicate that *Hpa* Waco9 overcomes recognition by RPP1, but not RPP5, through the deletion of *ATR1* from its genome.

### Expression pattern of Arabidopsis genes in compatible and incompatible interactions with *Hpa*

**Figure 4:**
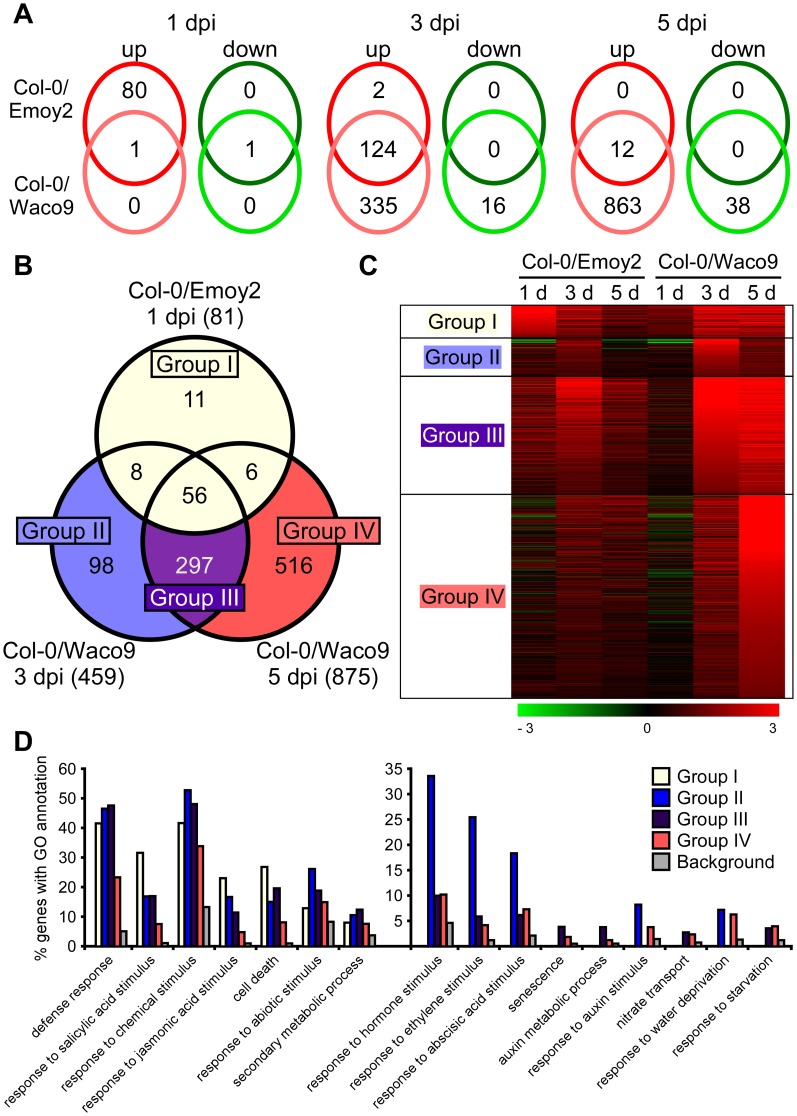
Arabidopsis genes differentially expressed after inoculation with *Hpa* Emoy2 and Waco9. (A) The number of Arabidopsis genes significantly upregulated or downregulated at 1, 3 and 5 dpi with *Hpa* Emoy2 and Waco9. (B) Assessment of overlap of genes significantly upregulated at 1 dpi with *Hpa* Emoy2 and at 3 and 5 dpi with *Hpa* Waco9, and classification into Group I (yellow), II (blue), III (purple) and IV (red). (C) Expression pattern of genes categorized into Group I, II, III and IV. The relative expression (in log_2_ ratios) is colored red for induction and green for repression as illustrated in the fold change color bars. (D) Percentage of genes with significantly enriched gene ontology (GO) terms in Group I (yellow), II (blue), III (purple) and IV (red), compared to the background (grey). Y-axis: percentage of genes that fall within each given GO annotation class.

We investigated Arabidopsis gene expression during infection with *Hpa* Emoy2 and Waco9. The expression of 24,559 Arabidopsis protein-coding genes (89.6% of the 27,416 protein-coding genes predicted in Arabidopsis TAIR10) was detected in at least one time point (Tables 2 and Dataset S1). Of these, 1,048 Arabidopsis genes showed significant changes in gene expression (FDR = 0.001) after inoculation with *Hpa* Emoy2 or Waco9 (Table S6). To reveal compatible- or incompatible-interaction-specific changes in gene expression, we determined the level of overlap of differentially expressed Arabidopsis genes between infections with *Hpa* Emoy2 and Waco9 (Figure 4A). We found that many genes were specifically upregulated at 1 dpi with *Hpa* Emoy2 (80 genes) and at 3 and 5 dpi with *Hpa* Waco9 (335 and 863 genes, respectively) (Figure 4A). The Arabidopsis genes upregulated at 1 dpi with *Hpa* Emoy2, but not Waco9, might be induced upon recognition by *RPP4* (i.e. ETI), while the genes upregulated in the interaction with *Hpa* Waco9, but not Emoy2, might be genes targeted by *Hpa* to enhance susceptibility. Therefore, we focused on upregulated Arabidopsis genes at 1 dpi with *Hpa* Emoy2 and at 3 and 5 dpi with *Hpa* Waco9, and categorized them into four groups: Group I, 81 upregulated Arabidopsis genes at 1 dpi with *Hpa* Emoy2; Group II, 98 upregulated Arabidopsis genes at only 3 dpi with *Hpa* Waco9; Group III, 297 upregulated Arabidopsis genes at both 3 and 5 dpi with *Hpa* Waco9; Group IV, 516 upregulated Arabidopsis genes at only 5 dpi with *Hpa* Waco9 (Figure 4B, C and Table S7). Interestingly, 86.4% of Arabidopsis genes in Group I (70 genes) were also upregulated at 3 and/or 5 dpi with *Hpa* Waco9. Gene Ontology (GO) term enrichment analysis showed that responses involved in disease resistance (e.g. defense response, GO:0006952; response to salicylic acid stimulus, GO:0009751) were significantly enriched in all Groups (Figure 4D left). These findings suggest that defense-related Arabidopsis genes upregulated at early time points in the incompatible interaction are similarly regulated at late time points in the compatible interaction. This is consistent with previous reports on expression profiling in Arabidopsis and *Hpa* interactions [26]–[29]. On the other hand, genes responsive to ET (GO:0009723) and hormones (GO:0009725), such as ABA (GO:0009737) and auxin (GO:0009733), were overrepresented in Group II, III and/or IV but absent in Group I, highlighting genes induced specifically during a compatible interaction (Figure 4D right). In these Groups, we also found overrepresentation of genes related to nitrate transport (GO:0015706), water deprivation (GO:0009414) and starvation (GO:0042594) (Figure 4D right).

### *Hpa* infection suppresses SA-inducible *PR1* expression in Arabidopsis

**Figure 5:**
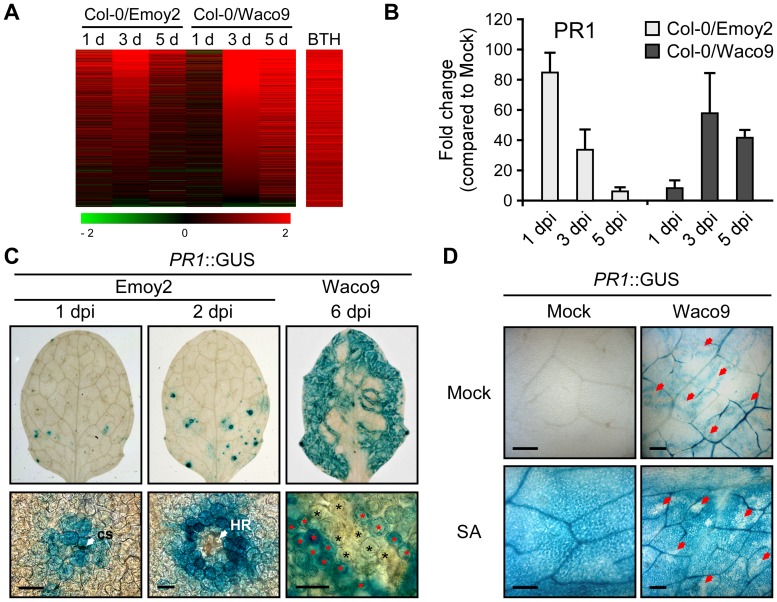
*Hpa* suppresses *PR1* expression induced by SA in infected cells. (A) Expression pattern of 871 BTH-inducible genes reported by Wang et al. (2006) [48] after inoculation with *Hpa* Emoy2 and Waco9. The relative expression (in log_2_ ratios) is colored red for induction and green for repression as illustrated in the fold change color bars. (B) Expression of *PR1* in Arabidopsis at 1, 3, and 5 dpi with *Hpa* Emoy2 and Waco9. The expression level was determined by qRT-PCR using specific primers for *PR1* and indicated as relative fold induction compared to water-treated samples (mock). Expression of *EF-1α* was used to normalize the expression value in each sample. Data are means ± SDs from three biological replicates. (C) GUS staining in three-week-old Arabidopsis leaves containing *PR1* promoter fused *GUS* (*PR1*::GUS) at 1 and 2 dpi with *Hpa* Emoy2 and at 6 dpi with *Hpa* Waco9. Lower images are magnified upper images. Black and red asterisks indicate *Hpa*-haustoriated and non-haustoriated mesophyll cells, respectively. cs, conidiospore. Scale bars = 40 µm. (D) GUS staining in *Hpa*-infected *PR1*::GUS lines 8 hours after treatment with SA (200 µM). The leaves at 4 dpi with *Hpa* Waco9 or spraying water (mock) were infiltrated with SA or water (mock). Red arrows indicate *Hpa*-haustoriated cells. Scale bars = 100 µm.

Defense-related Arabidopsis genes including SA-responsive genes were found to be upregulated not only at 1 dpi with *Hpa* Emoy2 but also at 3 and 5 dpi with *Hpa* Waco9 (Figure 4). Indeed, there was a positive correlation between these genes and genes upregulated by treatment with benzothiadiazole S-methylester (BTH; a functional analog of SA) [48] (Figure 5A and Table S8). At 1 dpi, BTH-inducible genes, such as *PR1*, were upregulated by inoculation with *Hpa* Emoy2, but not *Hpa* Waco9, whereas these genes were upregulated at 3 and 5 dpi with *Hpa* Waco9 (Figure 5A and B).

Recently, we reported the cell-specific expression pattern of *PR1* in a compatible interaction by infecting *PR1*::GUS lines with *Hpa* Waco9 [18]. *PR1*::GUS expression is suppressed in haustoriated cells, but not in non-haustoriated adjacent cells (Figure 5C) [18], but this could arise either via suppression of SA biosynthesis or SA responsiveness in these cells. To distinguish these possibilities, we investigated the effect of *Hpa* infection on SA- and BTH-inducible *PR1*::GUS expression. *PR1*::GUS lines at 4 dpi with *Hpa* Waco9 or mock infected were treated with SA, BTH or water. As expected, we observed GUS staining in non-infected *PR1*::GUS lines after treatment with SA and BTH (Figures 5D and S3). In *Hpa*-infected *PR1*::GUS lines, although GUS staining was observed in non-haustoriated cells after SA and BTH treatment, *Hpa*-haustoriated cells were not stained (Figures 5D and S3). These results suggest that *Hpa* suppresses the expression of *PR1* induced by treatment with SA and BTH. Thus, *Hpa* suppresses SA responsiveness by interfering with signaling, but not by promoting SA degradation.

We also investigated the cell-specific expression pattern of *PR1*::GUS in the incompatible interaction. GUS staining was observed in cells that *Hpa* Emoy2 had infected and the surrounding cells at 1 dpi, and observed in the cell layer surrounding cells in which HR cell death had occurred at 2 dpi (Figure 5C). These results are consistent with expression profiling data derived from whole *Hpa*-infected tissues (Figure 5A and B).

### A highly expressed *Hpa* effector, HaRxL62, suppresses responsiveness to SA

**Figure 6:**
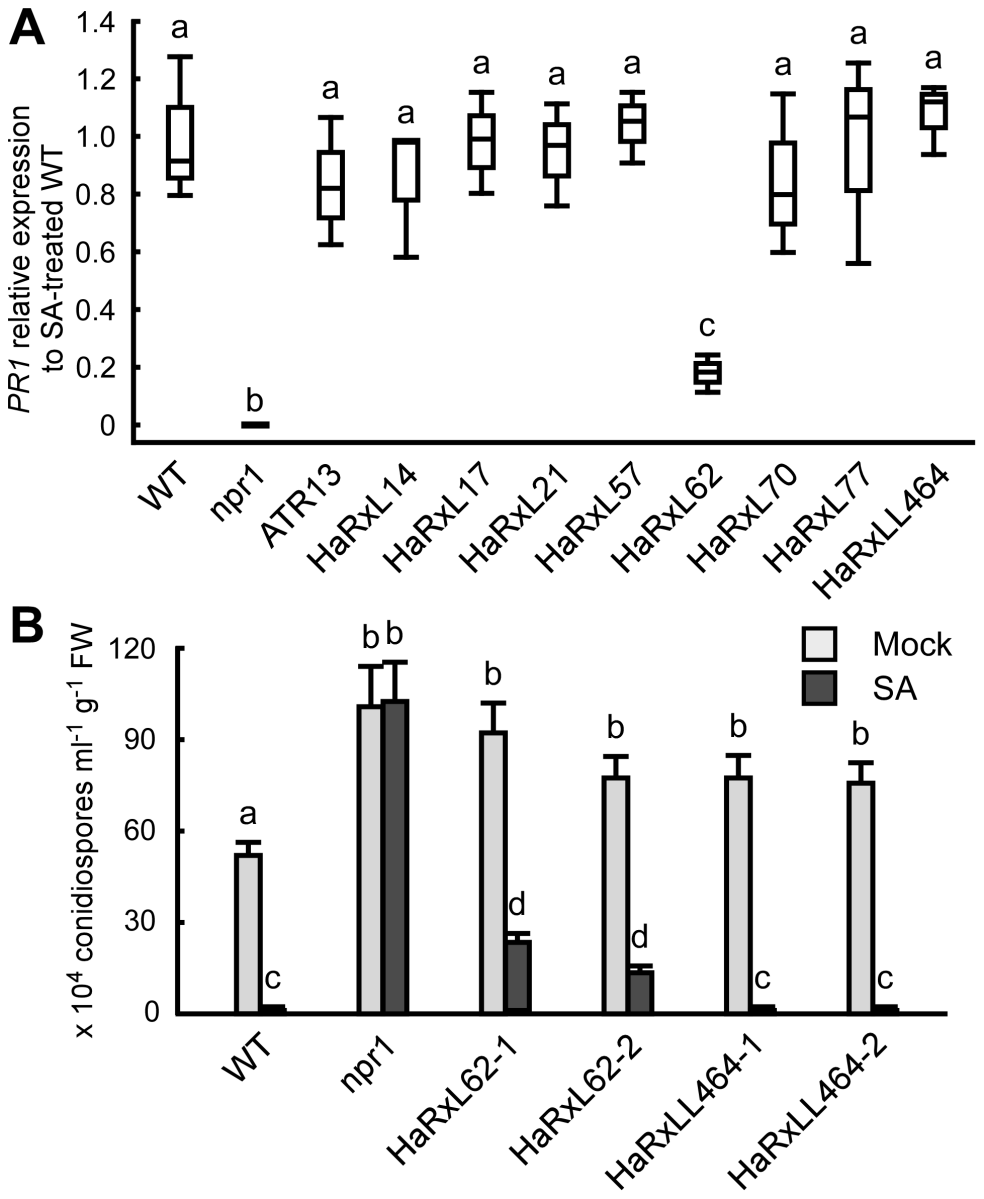
HaRxL62 reduces responsiveness to SA. (A) Expression level of *PR1* 8 hours after treatment with SA (100 µM) in ten-day-old Col-0 plants (WT), *npr1* mutants and transgenic lines expressing the indicated *Hpa* predicted effectors. The expression level was determined by qRT-PCR using specific primers for *PR1* and indicated as relative fold induction compared to the expression level in WT after SA treatment. Expression of *EF-1α* was used to normalize the expression value in each sample. Data are means from three biological replicates showing quantiles. Data analysis was carried using one-way ANOVA followed by Tukey's HSD (honestly significant difference). Genotypes showing significant differences (*p*<0.01) are marked with different alphabets (B) *Hpa* growth on three-week-old Col-0 plants (WT), *npr1* mutants and two independent transgenic lines expressing *HaRxL62* (HaRxL62-1 and HaRxL62-2) and *HaRxLL464* (HaRxLL464-1 and HaRxLL464-2) pretreated with SA (10 µM) or water (mock). The plants 24 hours after spray treatment with SA or water were inoculated with *Hpa* Waco9. Conidiospores were harvested and counted at 6 dpi. Different letters indicate significantly different values at *p*<0.05 (one-way ANOVA, Tukey's HSD).

Histochemical GUS analysis in *Hpa*-infected *PR1*::GUS lines showed that *Hpa* suppresses SA-inducible *PR1* expression specifically in the haustoriated cells into which RxLR effectors are delivered (Figure 5D). To identify *Hpa* effectors which participate in the suppression, the level of *PR1* expression after treatment with SA was checked in transgenic lines expressing *Hpa* predicted effectors and the SA-insensitive *npr1* mutants [49], as a positive control. Nine *Hpa* effector-expressing lines showed more susceptibility to *Hpa* compared to wild type (WT) Col-0 plants [8], [10] (Figure S4 and Table S9). *HaRxL62*-expressing lines showed a five-fold reduction in expression level of *PR1* compared to WT after SA treatment, whereas no significant reduction was observed in eight other *Hpa* effector-expressing lines, including *HaRxLL464*-expressing lines (Figure 6A). To evaluate the effect of HaRxL62 on *Hpa* growth after treatment with SA, WT plants, *npr1* mutants and *HaRxL62*- and *HaRxLL464*-expressing lines were treated with SA or water as a mock treatment and, 24 hours later, inoculated with *Hpa* Waco9 (Figure 6B). Although water-treated WT plants were susceptible to *Hpa* Waco9, no *Hpa* growth was observed in SA-treated WT plants. As expected, SA did not trigger resistance to *Hpa* in *npr1* mutants. In *HaRxLL464*-expressing plants treated with SA, essentially no *Hpa* spores were observed as observed for WT plants, whereas there were countable *Hpa* spores in *HaRxL62*-expressing plants treated with SA (Figure 6B), consistent with reduction in expression level of *PR1* after treatment with SA (Figure 6A). As shown in Figure 2A, *HaRxL62* was the second-highest expressed *Hpa* effector at 3 dpi. These results suggest that HaRxL62, a highly-expressed effector during infection, reduces responsiveness to SA.

## Discussion

A comprehensive understanding of host-pathogen interactions requires knowledge of the associated gene expression changes in both the host and the pathogen. However, in most cases, expression profiling has focused on either the host or the pathogen due to limitations and obstacles of older methods that involve microarrays [50]. In this study, using a high-throughput expression profiling method, EXPRSS [33], the transcriptomes of both Arabidopsis and *Hpa* in compatible and incompatible interactions were analyzed in parallel. With comparative genomics, we revealed that *Hpa* Waco9 evades *RPP1*-mediated resistance through deletion of cognate *AVR* gene *ATR1*. Histochemical analysis showed that *Hpa* suppresses SA-inducible *PR1* expression specifically in infected cells. Finally, we found a highly-expressed *Hpa* effector candidate involved in suppression of responsiveness to SA.

SA has been implicated as an important signal in plant immune signaling [51], [52]. For example, Arabidopsis *eds5/sid1* and *ics1/sid2* mutants in which SA levels are reduced [53], [54] are more susceptible to both virulent and avirulent forms of *P. syringae* and *Hpa* [51]. Expression profiling in Arabidopsis showed that SA-responsive genes including *PR1* are activated not only at early time points in the incompatible interaction but also at late time points in the compatible interaction (Figure 5A and B), consistent with previous reports [26]–[29]. Most recently, we reported that *Hpa* suppresses expression of *PR1*::GUS specifically in cells containing haustoria, into which host-translocated effectors are delivered, but not in non-haustoriated adjacent cells, which show high expression levels of *PR1*::GUS [18]. Here, we showed less *PR1*::GUS expression in *Hpa*-haustoriated cells after treatment with SA and BTH, indicating that *Hpa* interferes with the recognition of SA and/or downstream signaling after the recognition (Figure 5D). *HaRxL62*-expressing plants showed significant reduction in SA-induced expression of *PR1* and compromised resistance to *Hpa* after treatment with SA (Figure 6). HaRxL62 may make an important contribution to the virulence of *Hpa* because of its high expression levels during infection (Figure 2A). However, the suppression of SA-inducible resistance to *Hpa* in *HaRxL62*-expressing plants was moderate even though *HaRxL62*-expressing plants and *npr1* mutant plants showed comparable susceptibility to *Hpa* (Figure 6B). These findings suggest that HaRxL62 also targets other defense pathway(s) than the SA pathway and other *Hpa* effectors must also participate in suppression of responsiveness to SA. Anderson et al. (2012) [37] showed that HaRxL96 suppresses *PR1* expression, but not SA biosynthesis, induced by inoculation with an avirulent isolate of *Hpa*. HaRxL44 attenuates SA-dependent transcription through interfering with Mediator function by degrading MED19a, a transcriptional component involved in SA/JA crosstalk [18].

Our cell biology analysis also reveals a shortcoming of transcriptome analysis using whole tissues. We show that during *Hpa* infection, *PR1* is expressed in non-haustoriated adjacent cells, but not in haustoriated cells. We presume that recognition of diffusible PAMPs from *Hpa* leads to PTI, resulting in SA biosynthesis and *PR1* expression, and *Hpa* suppresses the responses in colonized cells by delivering effectors. Better methods are required for cell-type specific expression profiling specifically in haustoriated cells.

In addition to SA and JA, other phytohormones, such as ET, ABA and auxin, are also implicated in plant immunity [25]. ETHYLENE INSENSITIVE3 (EIN3) and ETHYLENE INSENSITIVE3-LIKE1 (EIL1), two closely related Arabidopsis transcription factors known to regulate the ET pathway, repress biosynthesis of SA by binding directly to the promoter of the SA biosynthetic gene *ICS1/SID2* [55]. Consistent with this, plants mutated in *EIN3/EIL1* and the key ET-signaling protein *EIN2* exhibit enhanced resistance to *P. syringae* [55] in spite of suppressed signaling of FLS2 which recognizes the bacterial PAMP flagellin [56]. Increased susceptibility to *P. syringae* and *Hpa* is observed in plants treated with ABA and in ABA over-accumulating plants, and vice versa in ABA-deficient mutants [57]–[59]. Similarly, elevated auxin signaling correlates with increase in susceptibility to *P. syringae* and *Hpa* [60]–[63]. Collectively, these findings suggest that ET, ABA and auxin behave as negative regulators of defense responses. Some bacterial effectors appear to target these signaling systems. Conditional expression of *P. syringae* effector AvrPtoB increases *in planta* ABA levels and enhances bacterial growth [64]. AvrBs3, a type three effector from *Xanthomonas campestris* pv. *vesicatoria*, induces auxin responsive genes, resulting in cell hypertrophy [65]. Our expression profiling in *Hpa*-infected Arabidopsis revealed overrepresentation of genes related to responses to ET (GO:0009723), ABA (GO:0009737) and auxin (GO:0009733) in Group II, III and/or IV, genes upregulated at 3 and/or 5 dpi with *Hpa* Waco9, but not at 1 dpi with *Hpa* Emoy2 (Figure 4). Consistent with this finding, previous expression profiling using microarrays in Arabidopsis Ler-0 inoculated with compatible (Cala2) and incompatible (Waco9, recognized by *RPP5*) *Hpa* isolates revealed that many compatible-specific genes are ABA responsive [28]. Interestingly, we also found that genes involved in nitrate transport (GO:0015706) were overrepresented in Group III and IV (Figure 4D). *Hpa* lacks genes for nitrate and nitrite reductases and a nitrate transporter [7], which is also true for another obligate biotrophic powdery mildew fungi [66]. Expression profiling in *Hpa* revealed 202 and 252 predicted effectors expressed at 3 and 5 dpi with *Hpa* Waco9, respectively (Table 1). Conceivably, some of these effectors target these phytohormone signaling and host nitrate transporter systems.

This study also showed expression patterns and levels of *Hpa* predicted effectors, which may help select *bona fide* virulence effectors. Indeed, the second-highest expressed *Hpa* effector at 3 dpi, HaRxL62, appears to enhance susceptibility at least in part by suppressing responsiveness to SA. In a previous screening of *Hpa* predicted effectors that enhance the virulence and/or that suppress PTI, HaRxL62 was selected as the most effective *Hpa* effector [8], [9]. HaRxL76, the highest-expressed *Hpa* effector at 3 dpi, was not in the list for our previous screenings. HaRxL76 and other highly-expressed *Hpa* predicted effectors will be investigated in future studies.

To evade recognition by cognate *R* genes, the majority of RxLR effector genes are subject to diversifying selection, resulting in a diverse set of effector alleles in the pathogen population [4], [5]. ATR1 and ATR13 have a high level of sequence polymorphism in the C-terminal regions that confer effector activity and are recognized by RPP1 and RPP13, respectively [35], [38]. In this study, we revealed that *ATR1* is deleted in *Hpa* Waco9 genome, resulting in loss of recognition by *RPP1* (Figure 3). Qutob et al. (2009) and (2013) [67], [68] reported that virulent strains of *Phytophthora sojae* escape detection by *R* gene *Rps3a* through silencing a cognate AVR effector *Avr3a*. In virulent pathogens, the effectors recognized by cognate *R* genes would be deleted and polymorphic like *ATR1* and *ATR13*, or not expressed like *Avr3a*. These possibilities can be evaluated by comparative genomics and transcriptomics.

In this study, we found overrepresentation of oomycete core element INR-FPR and two novel motifs, Motif I and II, in the promoter of *Hpa* predicted effectors (Figure 2). The INR-FPR motif is associated with higher levels of transcripts and pathogenesis-related genes including RxLR effectors in *P. infestans* [43]. Consistent with this, the genes with the INR-FPR motif were highly enriched for both *Hpa* predicted effectors and *P. infestans* RxLR effectors, especially effectors induced during infection referred to as “induced effectors”. On the other hand, we found association of Motif I and II with “induced effectors” and “non-induced effectors”, respectively, in *Hpa*, but not in *P. infestans*. While *Hpa* and *P. infestans* may have a common pre-initiation complex for transcription, there might be distinct regulatory mechanisms for specific gene expression, perhaps resulting from different lifestyles. Although the findings may be useful for predicting potential effectors in related oomycetes, it will be difficult to investigate functions of these motifs in *Hpa* because transformation of biotrophic oomycete pathogens is difficult.

Here, we explored gene expression changes in both Arabidopsis and *Hpa* simultaneously during infection using a high-throughput RNA sequencing method, EXPRSS [33]. Although we cannot rule out the possibility that differences in effector sets between *Hpa* Emoy2 and Waco9 confer distinct transcriptional changes in Arabidopsis genes during infection, expression profiling of both pathogen effector genes and host genes involved in immunity allows us to suggest distinct mechanisms of effector-mediated susceptibility. When stably expressed *in planta*, some *Hpa* effectors cause diverse developmental phenotypes, highlighting that the effectors might interfere with fundamental plant regulatory mechanisms [69]. Further comparative investigations of transcriptional changes in Arabidopsis genes between *Hpa* infections and effector(s)-expressing plants would be interesting. Recently, using a custom-designed combined pathogen and host whole-genome microarray, Jupe et al. (2013) [70] reported a simultaneous overview of gene expression changes in both *Phytophthora capsici* and its host tomato during the infection. In comparison to their approach using a custom microarray, our approach using EXPRSS can be more easily applied to host-pathogen interactions for which both host and pathogen genome sequences are available. This work opens the door towards transcriptome studies in infection biology that should help unravel pathogen infection strategies and the mechanisms by which host defense responses are overcome.

## Materials and Methods

### Plant material and growth

Arabidopsis accessions used in this study were obtained from the Nottingham Arabidopsis Stock Centre. Arabidopsis RIL 3860 and 3860:RPP1Nd were kindly provided by Jim L. Beynon, University of Warwick, UK [35], and Arabidopsis CW84 and CW84:RPP5Ler were from Bailey et al. (2011) [40]. *PR1*::GUS lines were from Caillaud et al. (2013) [18], and plants expressing *Hpa* predicted effectors other than *HaRxL62* were from Fabro et al. (2011) [8] and Caillaud et al. (2012) [10] (Table S9). A construct for expressing *HaRxL62 in planta* was generated by recombining the corresponding ORF from the signal peptide cleavage site cloned in pENTR/SD/D-TOPO (Invitrogen) into the Gateway destination binary vector pENS-StrepII-3×HA-GW under the control of *Cauliflower mosaic virus* 35S promoter [71]. The construct was transferred to *Agrobacterium tumefaciens* strain GV3101 (pMP90 RK) [72] and transformed into Arabidopsis accession Col-0 by the floral dipping method [73]. Primary transformants (T1) were selected on soil containing BASTA (Bayer CropScience, Wolfenbüttel, Germany) and checked for expression of HaRxL62 by Western blot analysis as described by Asai et al. (2008) [74]. The progeny of the T2 generation was observed and 3∶1 (BASTA-resistant/BASTA-susceptible) segregating lines were taken further. Homozygous lines were selected by examining the BASTA resistance of T3 seedlings. Two independent transgenic lines were analyzed.

For *Hpa*-inoculation assay, Arabidopsis plants were grown at 22°C and 60% humidity under a 10-h photoperiod and a 14-h dark period in environmentally controlled growth cabinets. For SA-induced *PR1* expression analysis, Arabidopsis plants were grown on 0.7% agar plates of MS medium at 22°C under a 16-h photoperiod and an 8-h dark period in environmentally controlled growth cabinets.

### Pathogen assays

For *Hpa* infection, Arabidopsis plants were spray-inoculated to saturation with a spore suspension of 5×10^4^ conidiospores/ml. Plants were covered with a transparent lid to maintain high humidity (90–100%) conditions in a growth cabinet at 16°C under a 10-h photoperiod until the day for sampling.

To evaluate hyphae growth and HR cell death, leaves inoculated with *Hpa* Emoy2 or Waco9 were stained with trypan blue as described by Asai and Yoshioka (2009) [75].

To evaluate conidiospore production, 5 pools of 3 plants for each Arabidopsis line were harvested in 1 ml of water. After vortexing, the amount of conidiospores released was determined using a haemocytometer.

### RNA extraction, cDNA synthesis and qRT-PCR

Total RNAs were extracted using TRI reagent (Sigma) and 1-bromo-3-chloropropane (Sigma) according to the procedure of the manufacturer. RNAs were precipitated with half volume of isopropanol and half volume of high salt precipitation buffer (0.8 M sodium citrate and 1.2 M sodium chloride). RNA samples were treated with DNaseI (Roche) and purified by RNeasy Mini Kit (Qiagen) according to the procedure of the manufacturers.

Total RNAs (3 µg) were used for generating cDNAs in a 20 µl volume reaction according to Invitrogen Superscript II Reverse Transcriptase protocol. The obtained cDNAs were diluted five times, and 1 µl were used for 10 µl qPCR reaction.

qPCR was performed in 10 µl final volume using 5 µl SYBR Green mix (Sigma), 1 µl diluted cDNAs, and primers. qPCR was run on the CFX96 Real-Time System C1000 thermal cycler (Biorad) using the following program: (1) 95°C, 3 min; (2) [95°C, 30 sec, then 60°C, 30 sec, then 72°C, 30 sec]×45, 72°C, 10 min followed by a temperature gradient from 55°C to 95°C. The relative expression values were determined using *EF-1α* (*At5g60390*) as a reference gene and the comparative cycle threshold method (2^−ΔΔCt^). Primers used for qPCR are listed in Table S10.

### *Hpa* Waco9 genome sequencing

Genomic DNA was extracted from *Hpa* Waco9 conidiospores using a Nucleon PhytoPure DNA extraction kit (GE Healthcare) according to the procedure of the manufacturer. A paired-end 400 bp insert size library was prepared and sequenced on Illumina Genome Analyzer II. The sequence reads were aligned in a paired end fashion to the *Hpa* Emoy2 v8.3 [7] using BWA [76]. Trailing nucleotides with a quality score of less than 10 were trimmed using the -q option. In order to maximize the number of aligned reads, unaligned reads were aligned using a more sensitive aligner, Stampy [77]. SAMtools [78] was used to generate a BAM file that enables visualization of the alignment with the Integrative Genomics Viewer [79], as seen in Figure 3C.

For correction of *Hpa* genome by Waco9 SNVs, genetic variations between *Hpa* Emoy2 and Waco9 were predicted using SAMtools [78]. *Hpa* Emoy2 v8.3 genome sequence [7] was corrected by substituting *Hpa* Waco9 SNVs, using a custom Perl script. Insertion and deletion variations were ignored. The sequence data have been deposited in NCBI's Short Read Archive (SRA) and are accessible through SRA accession number SRX493773.

### RNA sequencing

RNA sequencing was performed as described previously [33]. Purified double stranded cDNAs were subjected to Covaris shearing (parameters: intensity, 5; duty cycle, 20%; cycles/burst, 200; duration, 60 sec). The libraries were sequenced on Illumina Genome Analyzer II. The sequence data have been deposited in NCBI's Gene Expression Omnibus (GEO) and are accessible through GEO Series accession number GSE53641. Sequence reads to gene associations were carried out using the considerations described previously [33]. Quality-filtered libraries were aligned to the combined genome of Arabidopsis TAIR10 and *Hpa* Emoy2 v8.3 [7] using Bowtie version 0.12.8 [80]. Unaligned reads from previous step were aligned to the combined genome reference using Novoalign v2.08.03 (http://www.novocraft.com/). Remaining reads were aligned to transcript sequences of Arabidopsis Col-0 (ftp://ftp.Arabidopsis.org/home/tair/Sequences/blast_datasets/TAIR10_blastsets/TAIR10_cdna_20101214_updated) using Bowtie version 0.12.8 [80]. The reads with up to 10 reportable alignments or uniquely aligned reads were selected for downstream analysis. Differential expression analysis was performed using the R statistical language version 2.11.1 with the Bioconductor [81] package, edgeR version 1.6.15 [82] with the exact negative binomial test using tagwise dispersions.

### Identification of DNA motifs

For identifying *cis*-regulatory elements, 200 and 500 nt upstream of the start codon of coexpressed *Hpa* genes categorized into five groups as shown in Figure 2B and Table S3 were extracted from Waco9-SNVs-corrected v8.3 genome sequence using a custom Perl script. The sets of sequences extracted from genes categorized into “induced effectors” and “non-induced effectors” were searched separately using MEME version 4.9.1 (http://meme.nbcr.net/meme/cgi-bin/meme.cgi) [41]. MEME was run with minimum width of 6 and maximum width of up to 20 and zero or one per sequence was allowed.

The abundance of each motif found by MEME analysis in other groups was evaluated per individual motif using FIMO (http://meme.nbcr.net/meme/cgi-bin/fimo.cgi) [42] with a q-value cutoff 1e-4. Similarity to known motifs was assessed using TOMTOM (http://meme.nbcr.net/meme/cgi-bin/tomtom.cgi) [45] against the JASPAR database.

In *P. infestans* isolate T30-4, genes were categorized into five groups according to whether genes were significantly upregulated at 2 and 3 dpi in microarray data of Cooke et al. (2012) [46]. As described above, 200 and 500 nt upstream of the start codon of coexpressed *P. infestans* genes were extracted, and then the abundance of each motif was evaluated using FIMO [42].

### GO enrichment analysis

To investigate enrichment of specific gene ontologies in Arabidopsis genes categorized into four groups (Group I to IV) as shown in Figure 4D and Table S7, the Singular Enrichment Analysis was done with FDR = 0.05 using AgriGO (http://bioinfo.cau.edu.cn/agriGO/analysis.php).

### GUS staining

GUS activity was assayed histochemically with 5-bromo-4-chloro-3-indolyl-β-D-glucuronic acid (1 mg/ml) in a buffer containing 100 mM sodium phosphate pH 7.0, 0.5 mM potassium ferrocyanide, 0.5 mM potassium ferricyanide, 10 mM EDTA, 0.1% Triton. Arabidopsis leaves were vacuum-infiltrated with staining solution and then incubated overnight at 37°C in the dark. Destaining was performed in 100% ethanol followed by incubation in chloral hydrate solution. Stained leaves were observed using a Zeiss Axioplan 2 microscope (Jena, Germany).

### SA-induced *PR1* expression analysis

For SA-induced *PR1* expression analysis as shown in Figure 6A, ten-day-old plants grown on MS medium plates were used. The plants were equilibrated in water overnight, and water was changed for 100 µM SA (Sigma) solution in the morning. After 8 h of incubation with SA, the plants were quickly dried and flash-frozen in liquid nitrogen. Five plants per condition were used for RNA extraction.

### Accession numbers

Sequence data of 475 *Hpa* predicted effectors can be found in NCBI's GenBank data library under accession numbers described in Table S2.

## Supporting Information

Dataset S1 **The expression patterns of Arabidopsis genes.**
(XLSX)

Dataset S2 **The expression patterns of** ***Hpa*** **Emoy2 genes.**
(XLSX)

Dataset S3 **The expression patterns of** ***Hpa*** **Waco9 genes.**
(XLSX)

Figure S1 **Density plots of Arabidopsis and** ***Hpa*** **gene expression.** Gene expression measured as numbers of reads matched were created for all biological replicates. Density plots of Arabidopsis (A to C) and *Hpa* (D, E) genes were created for Mock (A), *Hpa* Emoy2 (B, D) and Waco9 (C, E) inoculation. For each replicate number of sense & antisense genes detected (n) and total number of reads assigned (Z) to genes were also presented.
(PDF)

Figure S2 **Resistance and susceptibility to** ***Hpa*** **Emoy2 and Waco9 in Arabidopsis accessions.** Resistance (R) and susceptibility (S) to *Hpa* Emoy2 and Waco9 in seven-day-old Arabidopsis Col-0, Nd-1, Ws-2, Ler-0 and Oy-0 plants. The plants inoculated with *Hpa* Emoy2 and Waco9 were photographed at 6 dpi.
(PDF)

Figure S3 ***Hpa*** **suppresses BTH-inducible** ***PR1*** **expression.** GUS staining in *Hpa*-infected *PR1*::GUS lines 8 hours after treatment with BTH (200 µM). The leaves at 4 dpi with *Hpa* Waco9 or spraying water (mock) were infiltrated with BTH or water (mock). Red arrows indicate *Hpa*-haustoriated cells. Scale bars = 100 µm.
(PDF)

Figure S4 **Expression of transgenes in transgenic lines expressing** ***Hpa*** **predicted effectors.** RNA was extracted from Arabidopsis Col-0 (Con) and transgenic lines expressing the indicated *Hpa* predicted effectors (trans) of three biological replicates. Expression of transgenes was checked by semi-quantitative RT-PCR using specific primers for the indicated *Hpa* predicted effectors. Equal loads of cDNA were monitored by amplification of constitutively expressed *EF-1α*.
(PDF)

Table S1 **Summary of transcriptome data in Arabidopsis inoculated with** ***Hpa*** **(comparison of uniquely mapped vs up to 10 matching read data).**
(XLSX)

Table S2 **The sequences and expression levels of** ***Hpa*** **predicted effectors.**
(XLSX)

Table S3 **The list of** ***Hpa*** **genes categorized.**
(XLSX)

Table S4 **Over-representation of motifs in coexpressed genes of** ***Hpa*** **and** ***P. infestans*** **.**
(XLSX)

Table S5 **The list of** ***Hpa*** **predicted effectors expressed in** ***Hpa*** **Emoy2 and/or Waco9.**
(XLSX)

Table S6 **The expression patterns of Arabidopsis genes differentially expressed after inoculation with** ***Hpa*** **Emoy2 and Waco9.**
(XLSX)

Table S7 **The list of Arabidopsis genes categorized into Group I, II, III and IV.**
(XLSX)

Table S8 **Correlation between BTH-responsive genes and differentially upregulated genes in** ***Hpa*** **-inoculated Arabidopsis.**
(XLSX)

Table S9 **Information for** ***Hpa*** **effector-expressing lines in which SA-inducible** ***PR1*** **expression was tested.**
(XLSX)

Table S10 **Primers used for qRT-PCR and semi-quantitative RT-PCR.**
(XLSX)
