# Supplementary figures and images for: Expression Profiling during Arabidopsis/Downy Mildew Interaction Reveals a Highly-Expressed Effector That Attenuates Responses to Salicylic Acid
Source: PLoS Pathog. 2014 Oct 16;10(10):e1004443. doi: 10.1371/journal.ppat.1004443 (PMC4199768; doi:10.1371/journal.ppat.1004443)

Figure S1

A

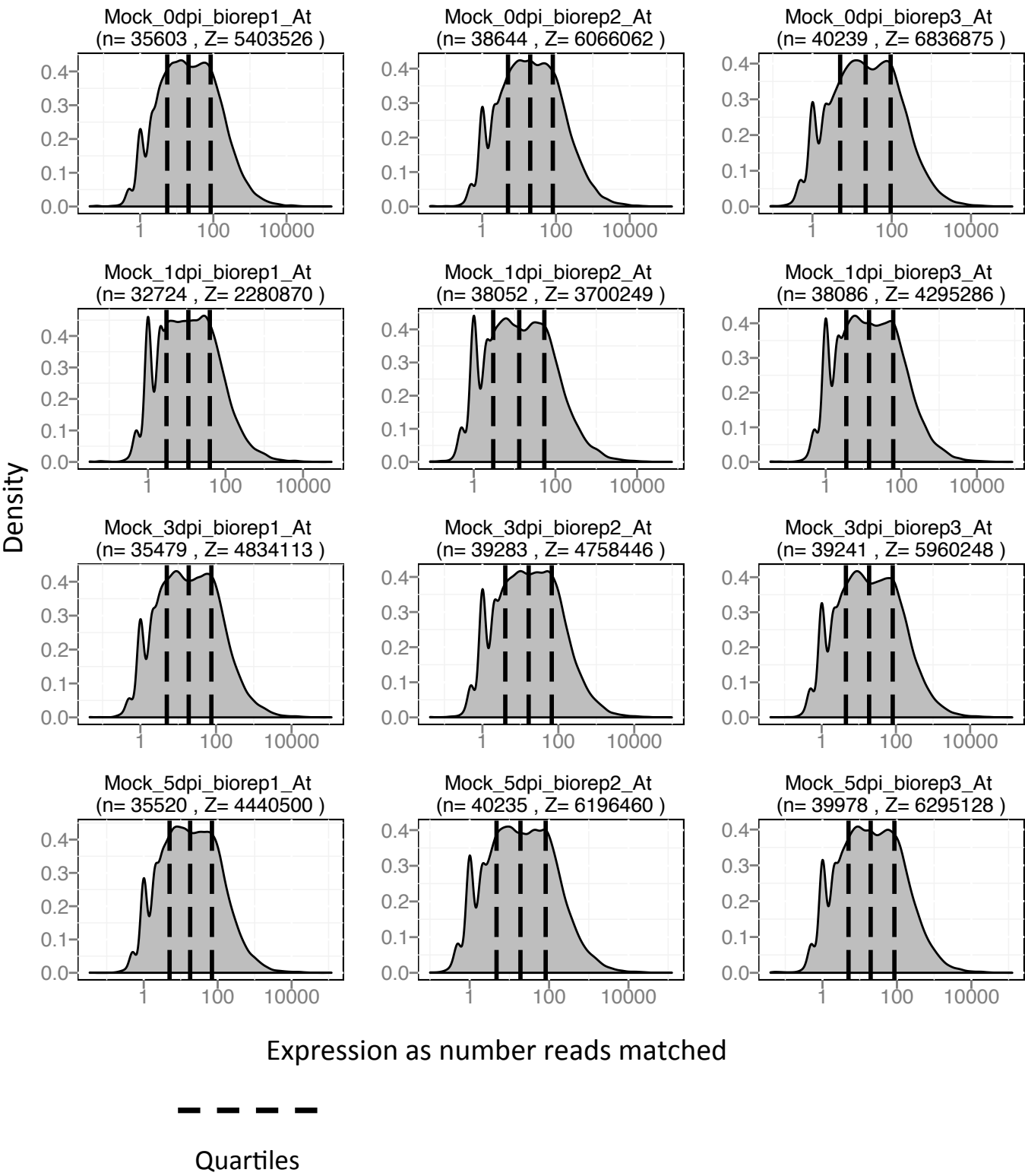

B

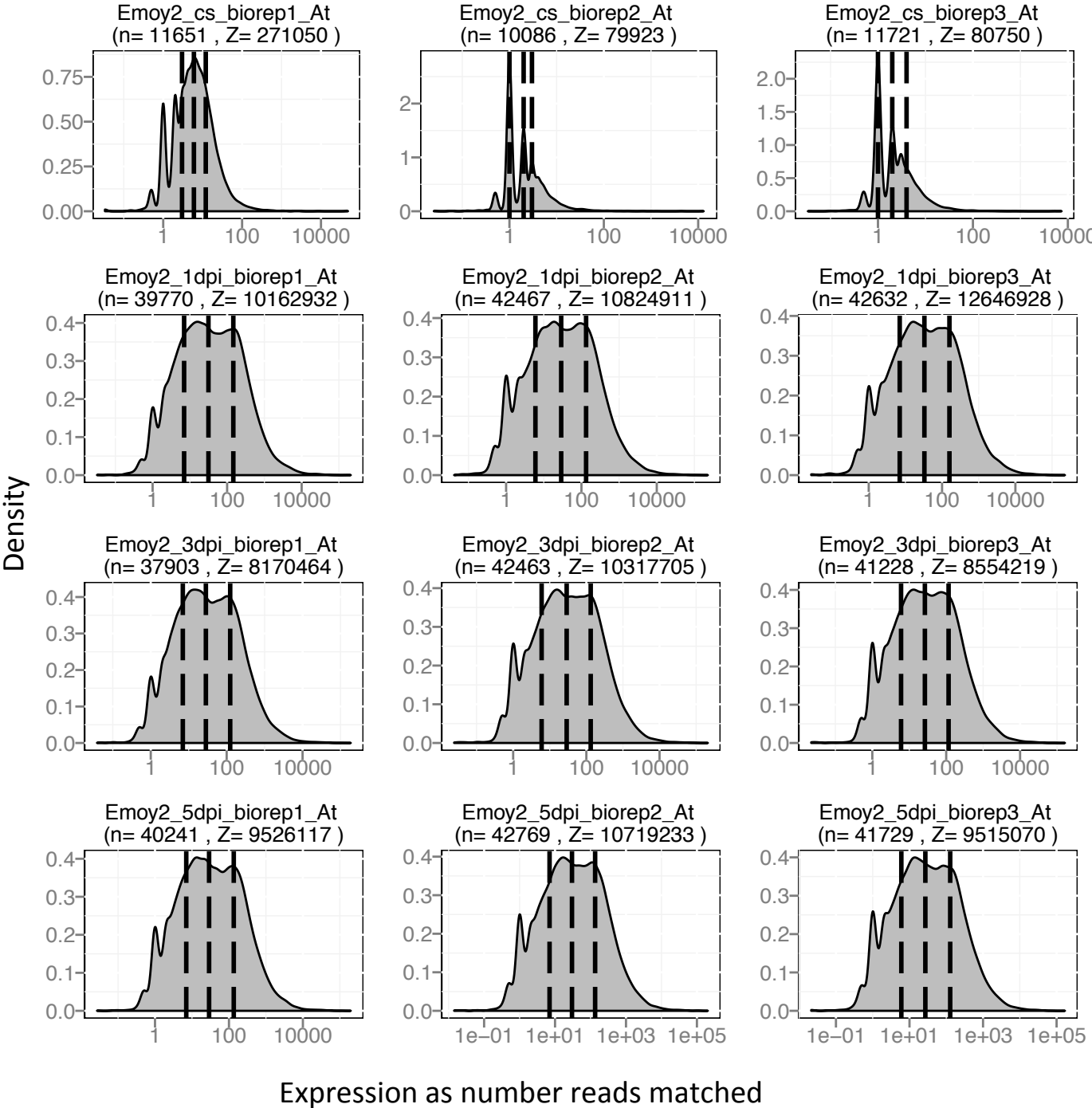

C

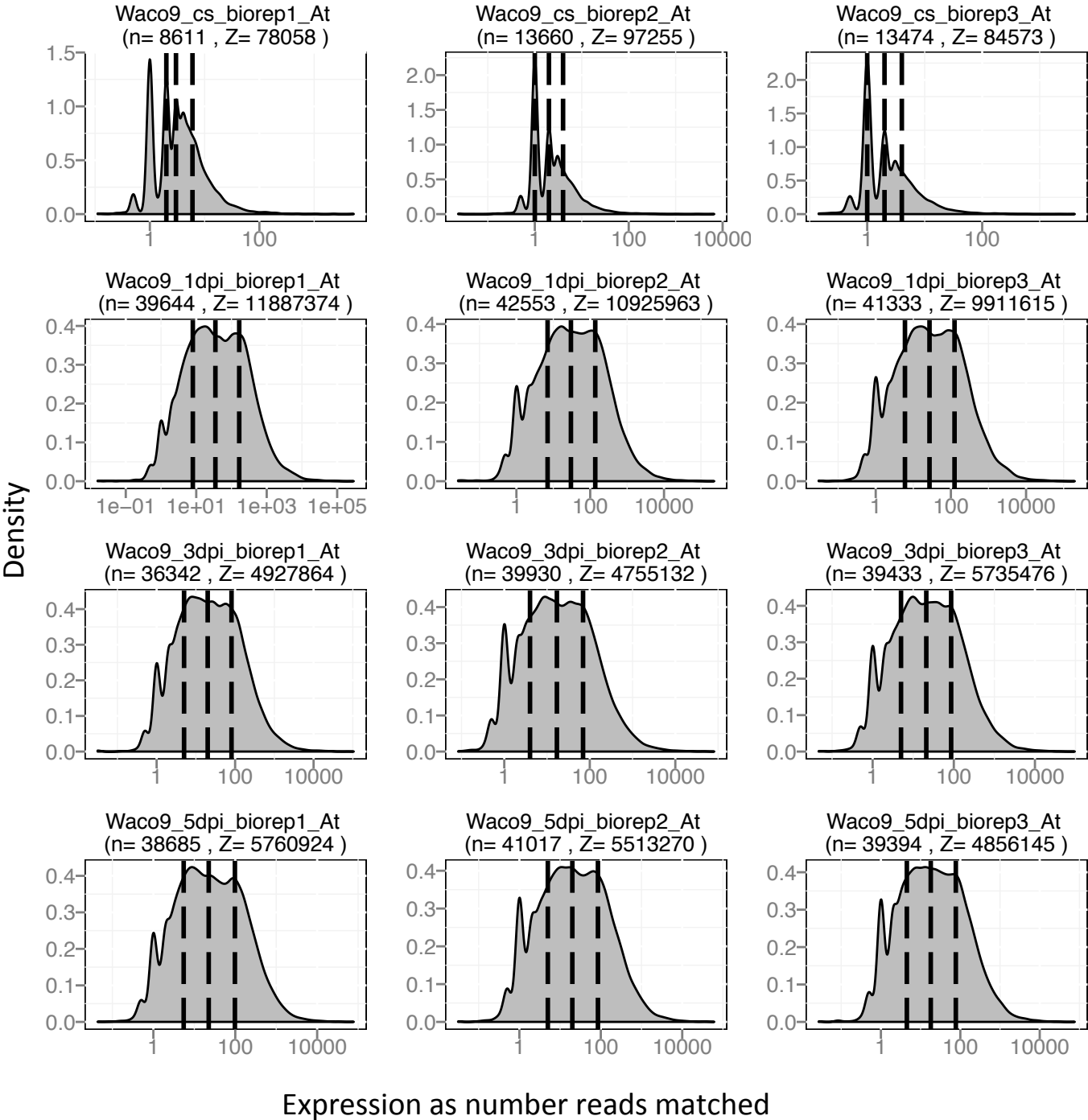

D

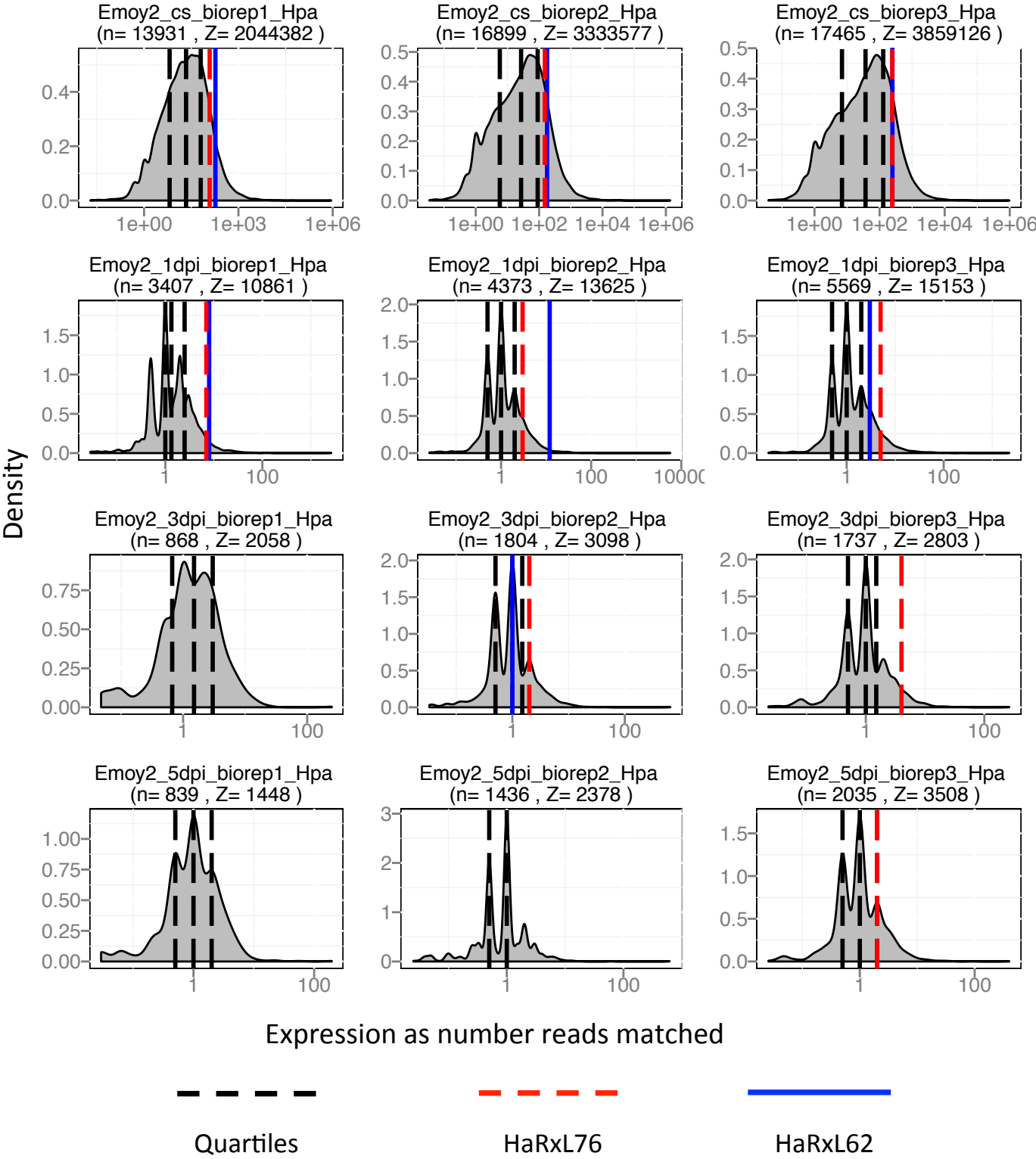

E

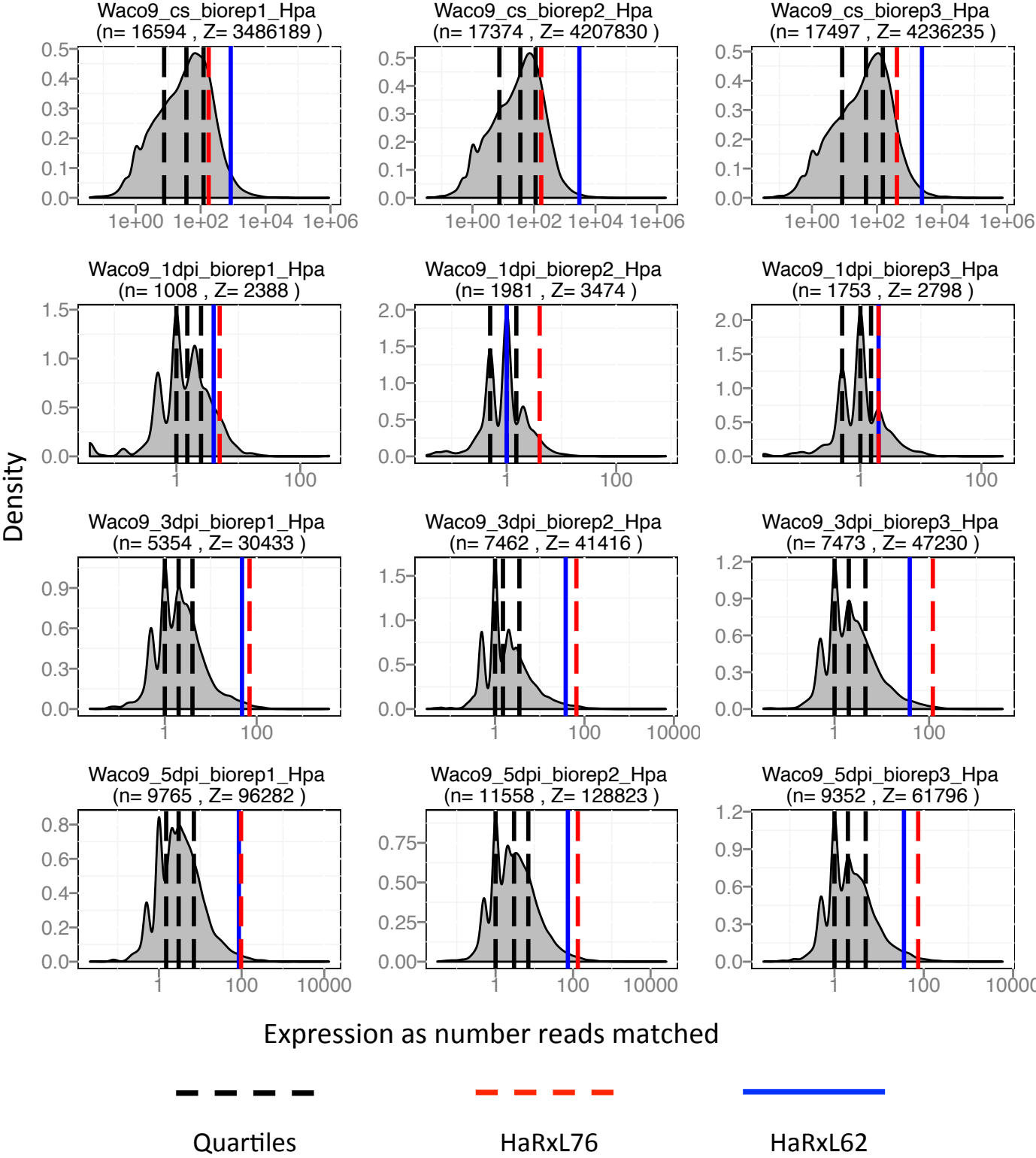

Supplement: Figure S1 — Density plots of Arabidopsis and Hpa gene expression. Gene expression measured as numbers of reads matched were created for all biological replicates. Density plots of Arabidopsis (A to C) and Hpa (D, E) genes were created for Mock (A), Hpa Emoy2 (B, D) and Waco9 (C, E) inoculation. For each replicate number of sense & antisense genes detected (n) and total number of reads assigned (Z) to genes were also presented. (PDF) [file ppat.1004443.s004.pdf]

Figure S2

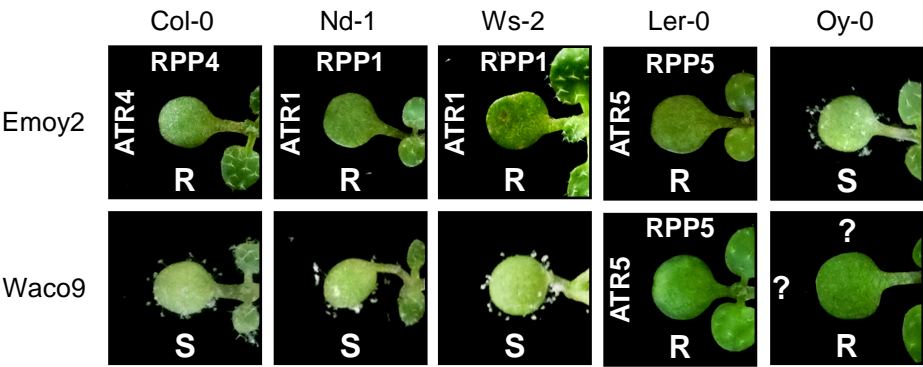

Supplement: Figure S2 — Resistance and susceptibility to Hpa Emoy2 and Waco9 in Arabidopsis accessions. Resistance (R) and susceptibility (S) to Hpa Emoy2 and Waco9 in seven-day-old Arabidopsis Col-0, Nd-1, Ws-2, Ler-0 and Oy-0 plants. The plants inoculated with Hpa Emoy2 and Waco9 were photographed at 6 dpi. (PDF) [file ppat.1004443.s005.pdf]

Figure S3

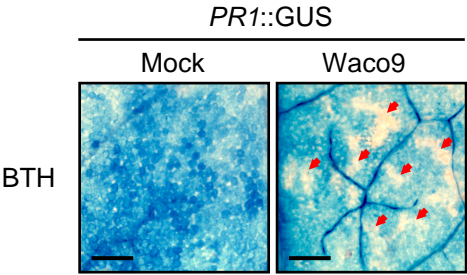

Supplement: Figure S3 — Hpa suppresses BTH-inducible PR1 expression. GUS staining in Hpa-infected PR1::GUS lines 8 hours after treatment with BTH (200 µM). The leaves at 4 dpi with Hpa Waco9 or spraying water (mock) were infiltrated with BTH or water (mock). Red arrows indicate Hpa-haustoriated cells. Scale bars = 100 µm. (PDF) [file ppat.1004443.s006.pdf]

Figure S4

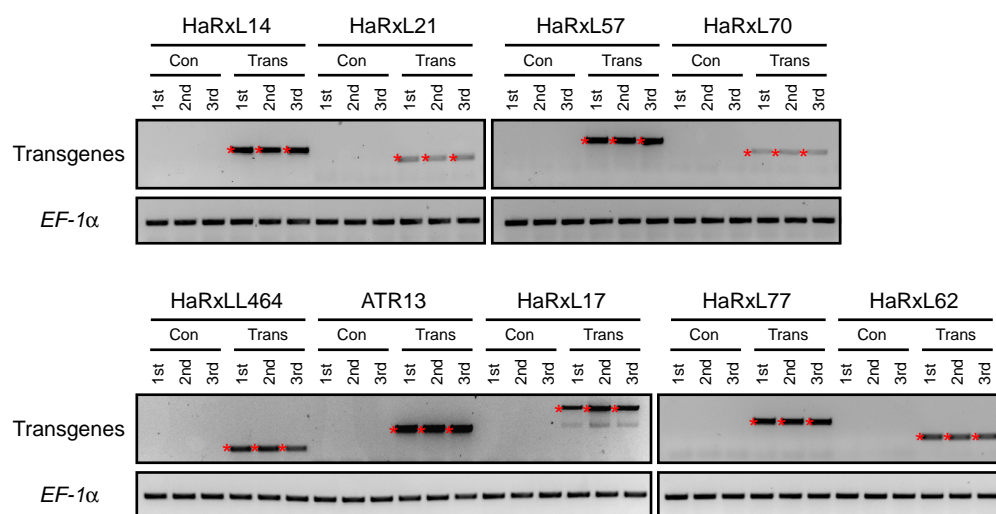

Supplement: Figure S4 — Expression of transgenes in transgenic lines expressing Hpa predicted effectors. RNA was extracted from Arabidopsis Col-0 (Con) and transgenic lines expressing the indicated Hpa predicted effectors (trans) of three biological replicates. Expression of transgenes was checked by semi-quantitative RT-PCR using specific primers for the indicated Hpa predicted effectors. Equal loads of cDNA were monitored by amplification of constitutively expressed EF-1α. (PDF) [file ppat.1004443.s007.pdf]
